# Predicted lean body mass trajectories, and cancer risk and cancer‐specific and all‐cause mortality: A prospective cohort study

**DOI:** 10.1002/jcsm.13370

**Published:** 2023-11-15

**Authors:** Chenan Liu, Qingsong Zhang, Tong Liu, Qi Zhang, Mengmeng Song, Guotian Ruan, Shiqi Lin, Ziwen Wang, Xin Zheng, Yue Chen, Heyang Zhang, Yizhong Ge, Hailun Xie, Jinyu Shi, Li Deng, Shouling Wu, Hanping Shi

**Affiliations:** ^1^ Department of Gastrointestinal Surgery, Department of Clinical Nutrition, Beijing Shijitan Hospital Capital Medical University Beijing China; ^2^ National Clinical Research Center for Geriatric Diseases, Xuanwu Hospital Capital Medical University Beijing China; ^3^ Key Laboratory of Cancer FSMP for State Market Regulation Beijing China; ^4^ Beijing International Science and Technology Cooperation Base for Cancer Metabolism and Nutrition Beijing China; ^5^ Department of General Surgery Kailuan General Hospital Tangshan China; ^6^ Department of Genetics Yale University School of Medicine New Haven Connecticut USA; ^7^ Cardiovascular Research Institute University of California San Francisco California USA; ^8^ Department of Cardiology Kailuan General Hospital Tangshan China

**Keywords:** Body composition, Predicted lean mass, Trajectory, Cancer risk, Mortality

## Abstract

**Background:**

Although many studies have investigated the association between body composition, cancer risk and mortality, predicting these risks through a single body composition measurement undoubtedly increases the limitations of the study. Few studies have explored the association between the trajectory of changes in body composition and the risk of cancer and death. We aimed to explore the association of predicted lean mass trajectories with cancer risk, cancer‐specific mortality and all‐cause mortality.

**Methods:**

The participants in this study were all from the Kailuan cohort, a prospective, periodic, resurvey cohort study initiated in 2006. Latent mixture modelling was used to identify predicted lean mass trajectories for 2006–2010. The hazard ratios (HRs) and 95% confidence intervals (95% CIs) of the Cox proportional hazard models were used to describe the association between predicted lean mass trajectories and cancer risk and cancer‐specific and all‐cause mortality during follow‐up (2010–2021).

**Results:**

A total of 44 374 participants (average age, 53.01 ± 11.41 years, 78.99% men and 21.01% women) were enrolled in this study. Five distinct trajectories were identified: low‐stable (*n* = 12 060), low‐increasing (*n* = 8027), moderately stable‐decreasing (*n* = 4725), moderately stable‐increasing (n = 8053) and high‐stable (*n* = 11 509). During the 11‐year follow‐up period, 2183 cancer events were recorded. After adjusting for age, predicted fat mass in 2010, sex, BMI, sedentary, physical activity, smoke, alcohol use, salt consumption, high‐fat diet, high‐sensitivity C‐reactive protein, serum creatinine, family history of tumour, hypertension, diabetes mellitus, compared with the low‐stable group, participants in the low‐increasing group (HR = 0.851, 95% CI, 0.748–0.969), moderately stable‐increasing group (HR = 0.803, 95% CI, 0.697–0.925) and high‐stable group (HR = 0.770, 95% CI, 0.659–0.901) had a lower cancer risk, but not in the moderately stable‐decreasing group (HR = 0.864, 95% CI, 0.735–1.015). Compared with the low‐stable group, the risk of cancer‐specific mortality was reduced by 25.4% (8.8–38.9%), 36.5% (20.3–49.4%) and 35.4% (17.9–49.2%), and the risk of all‐cause mortality was reduced by 24.2% (16.9–30.8%), 37.0% (30.0–43.2%) and 47.4% (41.0–53.1%) in the low‐increasing, moderately stable‐increasing group and high‐stable groups, respectively.

**Conclusions:**

Predicted lean mass trajectories may be closely associated with cancer risk and cancer‐specific and all‐cause mortality. Regular monitoring of body composition is necessary.

## Introduction

Cancer remains a major public health threat globally, and it is extremely important to identify the determinants of cancer incidence.^1^ Numerous studies have investigated the relationship between body mass index (BMI) and cancer risk, and some studies have shown that an increase in BMI is positively correlated with the incidence of colon and liver cancers, whereas it is negatively correlated with lung cancer risk.^2^, ^3^ However, there is controversy over whether there are still differences in cancer risk among different races and sexes at the same BMI level. Compared with a BMI of 22 kg/m^2^, the risk ratio of cancer for white adults with a BMI of 35 kg/m^2^ is 1.83, whereas that for black adults is 0.89.^4^ Even among participants of the same race with the same BMI, there are still significant differences in cancer risk.^5^ Although BMI is convenient to measure and use, it lacks a detailed assessment of body composition because an increase in either lean mass or fat mass (FM) can cause an increase in BMI. Therefore, relying solely on BMI to judge the risk of tumour occurrence may increase errors.

Song et al. showed that increased FM is associated with a higher risk of oesophageal and colon cancer.^6^ Another prospective study showed that FM versus fat‐free mass (FFM) based on bioelectrical impedance analysis influenced the risk of 16 common cancers, of which FFM was a stronger predictor.^7^ Even more so, studies have illustrated that, based on anthropometric equations, higher lean mass rather than FM is associated with a decreased risk of cancer,^8^ indicating the importance of body composition measurements. However, all studies share the common problem of judging the occurrence of tumours years or even decades later from only one body composition measurement. The changes in body composition are highly influenced by various factors, with one of the most significant being aging. As individuals age, mitochondrial function and the synthesis of bodily proteins weaken, while synthetic metabolism resistance increases, consequently, sarcopenia, characterized by the loss of lean mass and a decline in muscle function, has become one of the common chronic diseases associated with aging.^9^, ^10^ Furthermore, research indicated that individuals with metabolic disorders such as diabetes experience greater muscle loss compared with those without such conditions.^11^ Therefore, relying on a single measurement of lean mass during youth or a disease‐free period to predict outcomes over 5 or even 10 years undoubtedly diminishes the rigour and credibility of the study, as we are aware that aging and metabolic changes are ongoing processes. Therefore, this study aimed to construct trajectories of change in lean mass through a prospective cohort with periodic resurvey and explored the associations between the trajectories and participants' cancer risk, cancer‐specific and all‐cause mortality.

## Methods

### Study design and participants

All participants were from the Kailuan cohort, an ongoing prospective study in Tangshan, China, as previously described.^12^ Since 2006, Kailuan General Hospital and its 11 affiliated hospitals have conducted physical examinations and annual follow‐ups of Kailuan Group employees. The physical examination included laboratory tests, clinical measurements and questionnaires, and the follow‐up information included outpatient follow‐up reports, electronic medical records, death certificates from the Provincial Vital Statistics Offices (PVSO), and data entries in the Tangshan Medical Insurance System and Kailuan Social Security Information System. A total of 101 510 participants (81 110 men and 20 400 women) aged 18–98 years underwent their first physical examination and signed a written informed consent in 2006–2007.

**Figure 1:**
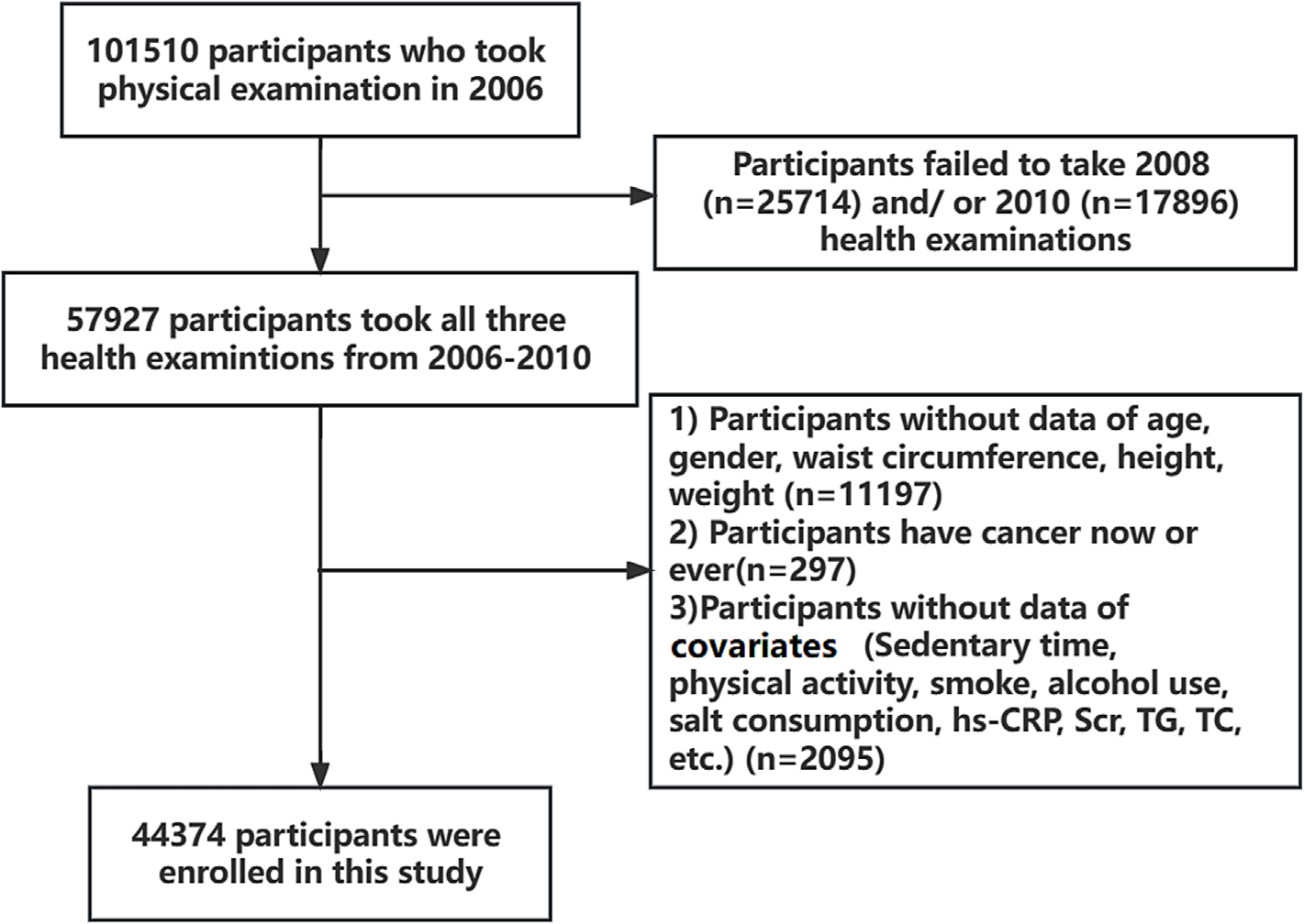
Flowchart of this study.

The predicted lean mass trajectories were constructed from three sets of physical examination data from 2006, 2008 and 2010 and used to predict the cancer risk of participants from 2010 to 2021. Therefore, patients with the following conditions were excluded: (1) who did not continuously participate in physical examinations from 2006 to 2010; (2) lacked cancer occurrence and survival data or patients with multiple cancers; (3) lacked sex, height, weight, waist circumference and age information; (4) presence or history of cancer; and (5) lacked covariate information. Participant recruitment is shown in Figure 1.

### Exposure and covariates

Predicted lean mass was calculated based on a sex‐, age‐ and race‐specific anthropometric equations that are widely validated and used and has high predictive power for both fat mass (*R* ^2^ = 0.90) and lean mass (*R* ^2^ = 0.91).^13^ Equations are presented in Methods S1.

Covariates recorded in 2010 included age, sex, BMI, sedentary lifestyle, physical activity (PA), smoking, alcohol consumption, salt intake, high‐fat diet, high‐sensitivity C‐reactive protein (hs‐CRP), serum creatinine (Scr), alanine aminotransferase (ALT), and family history of cancer, hypertension and diabetes mellitus, in addition to adjusting for participants' 2010 predicted fat mass to avoid the effects of fat. All covariate definitions are presented in Methods S2.

### Outcome

The primary outcome was cancer occurrence. The diagnosis of cancer was based on pathological, imaging, and clinical diagnoses in electronic medical records. In addition, to obtain cancer incidence information accurately, we integrated data from the PVSO, the Tangshan Medical Insurance System, and the Kailuan Social Security Information System. Diagnoses were coded using the International Classification of Diseases, 10th Revision (ICD‐10) (Methods S3). The follow‐up time was determined from the date of completion of the investigation in 2010 until the first occurrence of cancer, death, or the last follow‐up (31 December 2021), whichever occurred first.

The secondary outcomes were all‐cause and cancer‐specific mortality, defined as death from any cause, or cancer‐related death, or the status at last follow‐up (31 December 2021).

### Statistical analysis

All statistical analyses were conducted through SAS 9.3 (SAS Institute) or R version 4.2.2. Two‐sided *P* < 0.05 was considered statistically significant.

In our previous study, we found a significant sex‐related difference in lean mass.^10^ Therefore, in this study, we used the sex‐specific quintile of the predicted lean mass to build the trajectory model. Otherwise, women would be in the low lean mass trajectory group, while men would account for the main component in the high lean mass trajectory group, resulting in a sex bias. Latent mixture modelling was used to identify shared similar trajectories, which caused participants in the same group to have a similar trajectory of lean mass changes. This model was implemented using the PROC TRAJ program in SAS 9.3.^14^ The construction of the model was gradual. We first developed a model containing one trajectory, followed by two, three, four and up to five trajectory modes. The Bayesian information criterion (BIC) was used to evaluate the model fitness, with the smallest BIC indicating the most suitable model. Finally, we compared models with different functional forms and optimized them based on the significance of the cubic, quadratic and linear terms.

Variables conforming to a normal distribution are expressed as mean ± standard deviation (SD), and one‐way ANOVA was used for inter‐group comparison. Continuous variables with skewed distributions are represented as median and quartile range (IQR) and compared using a non‐parametric test. Categorical variables are expressed as rates, and the chi‐square test was used for comparisons between groups. Incidence rates are presented as per 1000 person‐years. First, we compared predicted lean mass trajectories, predicted lean mass in 2006, predicted lean mass in 2008, and predicted lean mass in 2010 through integrated discrimination improvement (IDI) and net reclassification improvement (NRI) to determine which approach is the best for assessing patients' prognosis. After the proportional risk assumption was satisfied, we used COX analysis to describe the association between different trajectories and cancer risk, as well as all cause and cancer‐specific mortality, and expressed them using hazard ratios (HR) and 95% confidence intervals (95% CI). Model 1 was adjusted for predicted fat mass only, while Model 2 was further adjusted for other covariates. In terms of digestive system cancer, we also adjusted for cirrhosis and hepatitis B. For gallbladder and extrahepatic cholangiocarcinomas, we additionally adjusted for the presence of gallstones and gallbladder polyps. For liver cancer, we additionally adjusted for cirrhosis, hepatitis B virus infection, gallstones and gallbladder polyps. These confounding factors have been shown to be related to the occurrence of cancer.^12^, ^15^, ^16^ We also conducted a subgroup analysis of specific cancer sites. Interaction analysis was performed using multiplicative interactions between the factors. We also supplemented the analysis of the association between baseline predicted lean mass in 2006 and cancer risk and used a restricted cubic spline (RCS) function to describe the nonlinear relationship between participants' predicted lean mass and cancer risk. Finally, sensitivity analysis was performed to verify the stability of the results. Sensitivity analyses excluded participants developing cancer within 1 year to avoid the possibility of causal inversion. As this was a rigorous prospective study, we excluded participants infected with hepatitis B virus, which has been proven to be closely related to digestive system cancers, and participants with a family history of cancer or abnormal Scr. At the same time, considering the potential collinearity among age, BMI and predicted lean mass, we included age^2^ and BMI^2^ as covariates in the model to control for the non‐linear relationships with age/BMI and the outcomes of interest. During the follow‐up process, covariates may change over time, and death as a competitive event may have affected the accuracy of the results. Therefore, we further adjusted for time‐varying variables and repeated our analysis using the Fine and Gray model, which can explain the bias caused by death as a competing risk.

## Results

**Figure 2:**
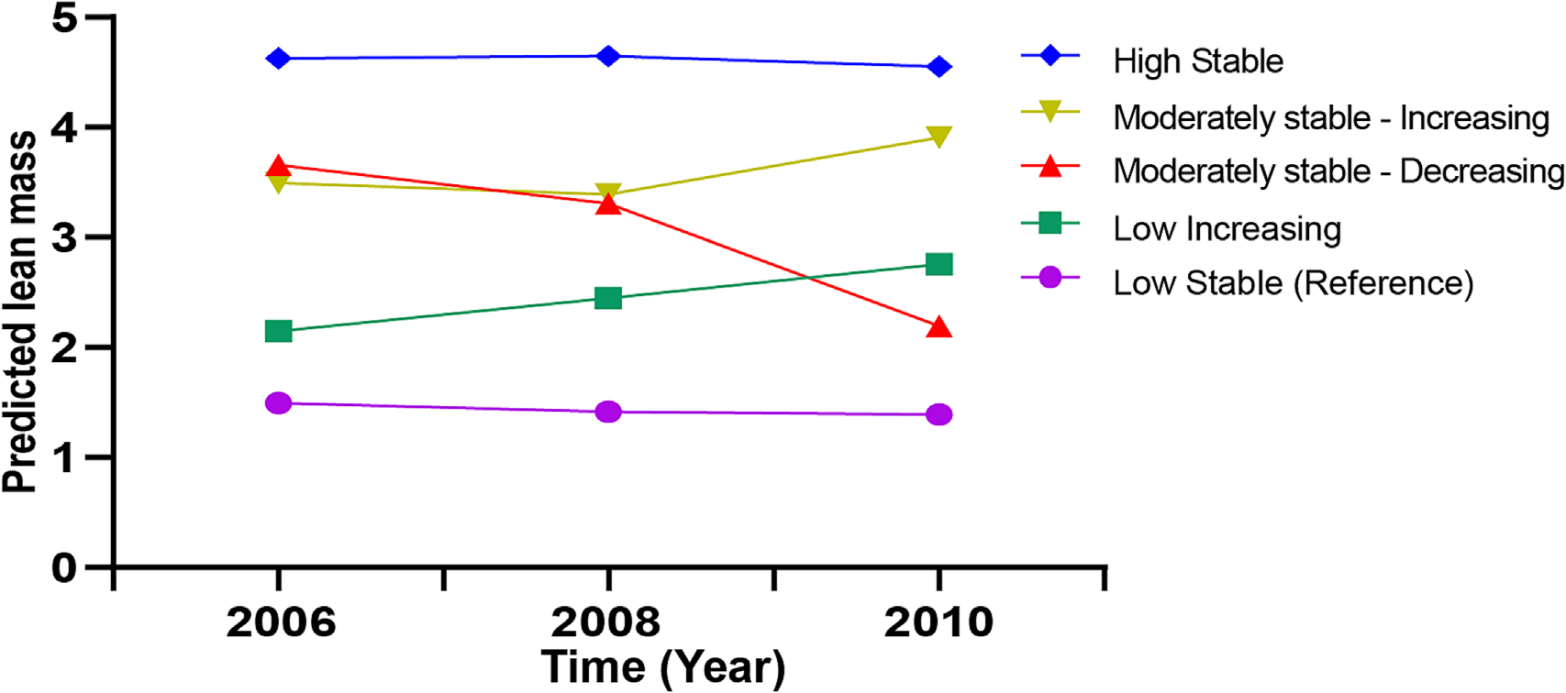
Predicted lean mass trajectory in participants during 2006–2010.

A total of 44 374 adults participated in this study, with an average age of 53.01 (11.41) years, and 35 049 (78.99%) participants were men. During the 11‐year follow‐up period, 2183 cancer events were recorded. Based on the participants' predicted lean mass from 2006 to 2010, we identified five trajectories: low‐stable (*n* = 12 060), low‐increasing (*n* = 8027), moderately stable‐decreasing (*n* = 4725), moderately stable‐increasing (*n* = 8053) and high‐stable (*n* = 11 509) (Figure 2). The baseline characteristics of the participants in 2006 according to the trajectory determined from 2006 to 2010 are shown in Table 1. Compared with the low‐stable group, the participants in the high‐stable group were younger, had a higher BMI and waist circumference, and had higher CRP, Scr and ALT levels. In addition, sedentary behaviour, regular PA, smoking, drinking, salt intake, hypertension, diabetes and other factors differed among the five groups.

**Table 1:** Baseline characteristics of participants based on predicted lean mass trajectory pattern

Characteristics		Low stable	Low increasing	Moderately stable: Decreasing	Moderately stable: Increasing	High stable	*P*
*N*		12 060	8027	4725	8053	11 509	
Age (year)		55.80 (12.35)	53.37 (11.83)	53.74 (11.08)	52.18 (11.36)	50.08 (11.20)	<0.001
Age (%)	<45	2148 (17.8)	1770 (22.1)	917 (19.4)	1993 (24.7)	3484 (30.3)	<0.001
	45–54	3895 (32.3)	2833 (35.3)	1753 (37.1)	2836 (35.2)	4182 (36.3)	
	55–64	3252 (27.0)	2151 (26.8)	1355 (28.7)	2235 (27.8)	2844 (24.7)	
	≥65	2765 (22.9)	1273 (15.9)	700 (14.8)	989 (12.3)	999 (8.7)	
Sex (%)	Men	9516 (78.9)	6250 (77.9)	3926 (83.1)	6341 (78.7)	9016 (78.3)	<0.001
Height (cm)		163.84 (6.54)	166.35 (6.31)	168.36 (5.90)	168.67 (6.23)	169.72 (6.60)	<0.001
Weight (kg)		60.06 (6.53)	65.56 (6.48)	73.88 (7.54)	72.73 (7.18)	80.51 (7.93)	<0.001
BMI (kg/m^2^)		22.41 (2.41)	23.73 (2.42)	26.12 (2.97)	25.61 (2.60)	28.17 (2.95)	<0.001
BMI (%, kg/m^2^)	<18.5	9046 (75.0)	4377 (54.5)	1018 (21.5)	2077 (25.8)	683 (5.9)	<0.001
	10.5–23.9	2832 (23.5)	3331 (41.5)	2742 (58.0)	4709 (58.5)	5136 (44.6)	
	≥24	182 (1.5)	319 (4.0)	965 (20.4)	1267 (15.7)	5690 (49.4)	
Waist circumference (cm)		81.00 [75.50, 87.00]	85.00 [80.00, 91.00]	85.00 [80.00, 91.00]	88.00 [82.00, 94.00]	91.00 [85.00, 97.00]	<0.001
Sedentary time (%, h)	<8	9358 (77.6)	6177 (77.0)	3700 (78.3)	6150 (76.4)	8751 (76.0)	0.005
	≥8	2702 (22.4)	1850 (23.0)	1025 (21.7)	1903 (23.6)	2758 (24.0)	
Regular physical activity (%)	Yes	1815 (15.0)	1072 (13.4)	668 (14.1)	1132 (14.1)	1597 (13.9)	0.011
Smoke (%)	Yes	4219 (35.0)	2887 (36.0)	1627 (34.4)	2835 (35.2)	3887 (33.8)	0.025
Alcohol consumer (%)	Yes	4364 (36.2)	3140 (39.1)	1751 (37.1)	3184 (39.5)	4502 (39.1)	<0.001
Salt consumption (%, g/day)	<6	1151 (9.5)	752 (9.4)	450 (9.5)	734 (9.1)	989 (8.6)	<0.001
	6–10	9757 (80.9)	6458 (80.5)	3772 (79.8)	6431 (79.9)	9211 (80.0)	
	>10	1152 (9.6)	817 (10.2)	503 (10.6)	888 (11.0)	1309 (11.4)	
Family history of tumour (%)	Yes	396 (3.3)	309 (3.8)	174 (3.7)	313 (3.9)	447 (3.9)	0.082
Triglyceride (mmol/L)		1.11 [0.79, 1.64]	1.21 [0.84, 1.81]	1.33 [0.97, 2.03]	1.34 [0.95, 2.07]	1.54 [1.08, 2.33]	<0.001
Total cholesterol (mmol/L)		4.88 [4.24, 5.56]	4.90 [4.25, 5.54]	4.94 [4.29, 5.59]	4.91 [4.28, 5.59]	4.93 [4.30, 5.61]	<0.001
hs‐CRP (mg/L)		0.60 [0.20, 1.93]	0.61 [0.23, 1.73]	0.71 [0.29, 1.90]	0.71 [0.29, 1.90]	0.88 [0.34, 2.10]	<0.001
Serum creatinine (μmoI/L)		85.00 [73.00, 99.00]	86.40 [74.00, 100.70]	92.00 [77.70, 107.50]	88.50 [75.70, 102.80]	91.00 [77.00, 106.00]	<0.001
ALT (μ/L)		16.20 [12.00, 22.00]	18.00 [13.00, 24.00]	19.00 [14.00, 25.00]	19.00 [13.40, 26.00]	20.00 [14.00, 27.00]	<0.001
Tbil (μmol/L)		12.30 [9.80, 15.30]	12.10 [9.80, 15.10]	11.90 [9.90, 14.80]	12.20 [9.90, 15.00]	12.10 [9.70, 14.90]	0.007
High‐fat diet (%)	Rare	1186 (9.8)	699 (8.7)	389 (8.2)	652 (8.1)	882 (7.7)	<0.001
	Occasional	9914 (82.3)	6657 (83.0)	3913 (82.8)	6653 (82.7)	9366 (81.4)	
	Frequent	952 (7.9)	665 (8.3)	422 (8.9)	742 (9.2)	1256 (10.9)	
Hypertension (%)	Yes	3991 (33.1)	2631 (32.8)	2104 (44.5)	3054 (37.9)	5319 (46.2)	<0.001
Diabetes mellitus (%)	Yes	813 (6.7)	510 (6.4)	459 (9.7)	566 (7.0)	1002 (8.7)	<0.001
Predicted lean mass 2006 (kg)		42.86 (6.10)	45.63 (6.16)	52.09 (7.02)	50.04 (7.07)	56.10 (8.50)	<0.001
Predicted lean mass 2008 (kg)		42.53 (5.83)	46.64 (6.44)	50.45 (7.23)	49.62 (6.75)	55.67 (8.31)	<0.001
Predicted lean mass 2010 (kg)		42.66 (5.79)	47.96 (6.58)	46.41 (5.51)	51.85 (7.19)	55.32 (8.17)	<0.001
Predicted fat mass 2010 (kg)		16.41 (4.58)	19.02 (4.90)	20.47 (4.65)	21.87 (5.05)	25.86 (5.59)	<0.001

Table S1 indicated that, compared with predicted lean mass trajectories, the predictive abilities of predicted lean mass in 2006, 2008 and 2010 were all worse for cancer risk, all‐cause mortality and cancer‐specific mortality.

There was a significant association between predicted lean mass and cancer risk (Table 2). Compared with the low‐stable group, participants in the low‐increasing group (HR = 0.851, 95% CI, 0.748–0.969), moderately stable‐increasing group (HR = 0.803, 95% CI, 0.697–0.925) and high‐stable group (HR = 0.770, 95% CI, 0.659–0.901) had a lower cancer risk. Interestingly, although the average predicted lean mass of participants in the moderately stable‐decreasing group was higher than that in the low‐stable group, there was no significant difference in cancer risk between the two groups (HR = 0.864, 95% CI, 0.735–1.015). We further distinguished cancer types to clarify the association between predicted lean mass trajectory and specific‐site cancer risk (Table S2). The results indicated that different trajectories of changes were associated with changes in the risk of digestive system cancers (gastric and colorectal cancers) and lung cancer. In terms of digestive system cancers, compared with the low‐stable group, the cancer risk of the other four groups was reduced, especially in the high‐stable group, where the HR for cancer risk was 0.614 (95% CI, 0.453–0.833). For lung cancer, only participants in the moderately stable‐increasing group (HR = 0.688, 95% CI, 0.521–0.908) and high‐stable group (HR = 0.683, 95% CI, 0.504–0.926) had a lower cancer risk.

**Table 2:** Association of predicted lean mass trajectories with cancer risk

Trajectory pattern	IR^a^	Case/total	Model 1^b^		Model 2^c^	
	4.72	2183/44 374	HR (95% CI)	*P*	HR (95% CI)	*P*
Low stable	5.19	652/12 060	Ref.		Ref.	
Low increasing	4.52	378/8027	0.805 (0.708, 0.915)	<0.001	0.851 (0.748, 0.969)	0.014
Moderately stable: Decreasing	4.69	231/4725	0.812 (0.696, 0.947)	0.008	0.864 (0.735, 1.015)	0.075
Moderately stable: Increasing	4.45	373/8053	0.735 (0.642, 0.842)	<0.001	0.803 (0.697, 0.925)	0.002
High stable	4.48	549/11 509	0.683 (0.595, 0.784)	<0.001	0.770 (0.659, 0.901)	<0.001

^a^ Incidence rate were presented as per 1000 person‐years.

^b^ Model 1 was adjusted for age and predicted fat mass in 2010.

^c^ Model 2 was adjusted for age, predicted fat mass in 2010, gender, BMI, sedentary, physical activity, smoke, alcohol use, salt consumption, high‐fat diet, hs‐CRP, Scr, family history of tumour, hypertension, diabetes mellitus.

Subsequently, we conducted a stratified analysis of the factors that may affect lean mass, and the results showed that the relationship between lean mass and the risk of whole cancer species was influenced by the interaction of covariates such as sex, age, regular PA, smoking and alcohol consumption (Table 3). This was more pronounced among men, older people, and participants with irregular PA, smoking and alcohol consumption. In addition, the relationship between predicted lean mass, digestive system cancers, and lung cancer was also influenced by covariates such as sex, age, regular PA, obesity, smoking and alcohol consumption (Tables S3 and S4).

**Table 3:** Subgroup analyses for the hazard ratio of cancer according to trajectories of predicted lean mass from 2006 to 2010

	*N*	Low stable	Low increasing	Moderately stable: Decreasing	Moderately stable: Increasing	High stable	*P* for interaction
Gender							<0.001
Women	9325	Ref.	1.135 (0.850, 1.515)	0.770 (0.499, 1.189)	1.047 (0.765, 1.433)	1.077 (0.753, 1.541)	
Men	35 046	Ref.	0.786 (0.679, 0.909)	0.885 (0.743, 1.056)	0.759 (0.647, 0.888)	0.714 (0.602, 0.848)	
Age (years)							0.043
<45	10 312	Ref.	0.893 (0.552, 1.446)	0.716 (0.365, 1.389)	0.831 (0.505, 1.377)	0.604 (0.341, 1.068)	
≥45	34 062	Ref.	0.855 (0.747, 0.979)	0.887 (0.752, 1.047)	0.859 (0.766, 0.978)	0.837 (0.814, 0.968)	
Regular PA							0.028
No	38 090	Ref.	0.853 (0.740, 0.983)	0.860 (0.720, 1.028)	0.781 (0.669, 0.912)	0.743 (0.628, 0.878)	
Yes	6284	Ref.	0.822 (0.598, 1.129)	0.949 (0.651, 1.383)	0.961 (0.692, 1.336)	1.025 (0.716, 1.467)	
Sedentary time							0.940
<8	34 136	Ref.	0.842 (0.728, 0.974)	0.884 (0.738, 1.058)	0.770 (0.657, 0.902)	0.791 (0.668, 0.937)	
≥8	10 238	Ref.	0.861 (0.647, 1.145)	0.836 (0.581, 1.203)	0.962 (0.713, 1.297)	0.762 (0.542, 1.071)	
Obesity							0.241
No	17 201	Ref.	0.786 (0.663, 0.932)	0.991 (0.748, 1.313)	0.777 (0.617, 0.977)	0.649 (0.443, 0.971)	
Yes	27 173	Ref.	1.008 (0.812, 1.253)	0.941 (0.754, 1.176)	0.924 (0.753, 1.134)	0.879 (0.717, 1.077)	
Smoke							0.018
No	28 919	Ref.	0.744 (0.601, 0.923)	0.875 (0.674, 1.135)	0.716 (0.565, 0.907)	0.710 (0.549, 0.917)	
Yes	15 455	Ref.	0.912 (0.775, 1.074)	0.869 (0.707, 1.068)	0.848 (0.729, 1.033)	0.824 (0.682, 0.995)	
Alcohol use							0.028
No	27 433	Ref.	0.808 (0.686, 0.952)	0.858 (0.702, 1.048)	0.761 (0.599, 0.967)	0.708 (0.547, 0.917)	
Yes	16 941	Ref.	0.906 (0.732, 1.120)	0.907 (0.692, 1.189)	0.835 (0.702, 0.992)	0.829 (0.688, 0.999)	
hs‐CRP							0.646
<2	10 909	Ref.	0.797 (0.622, 1.002)	0.726 (0.533, 0.989)	0.712 (0.546, 0.928)	0.711 (0.537, 0.941)	
≥2	33 465	Ref.	0.874 (0.780, 1.018)	0.951 (0.787, 1.149)	0.859 (0.728, 1.014)	0.829 (0.693, 0.999)	

We analysed the association between predicted lean mass and cancer risk in 2006. The RCS showed an inverted J‐shaped relationship between the predicted lean mass and cancer risk (Figure S1). Compared with quintile 1, the risk of oesophageal cancer (HR = 0.285, 95% CI, 0.111–0.733), gastric cancer (HR = 0.389, 95% CI, 0.203–0.745), digestive system cancers (HR = 0.522, 95% CI, 0.383–0.710) and lung cancer (HR = 0.631, 95% CI, 0.450–0.886) in quintile 5 participants showed a decreasing trend (Table S5).

At the same time, to emphasize the role of body composition, we also divided patients into four groups based on the median values of predicted lean mass and predicted fat mass (Table S6). The results indicated that compared with patients with low fat and low lean mass, participants with low fat and high lean mass had the lowest cancer risk (HR = 0.838, 95% CI, 0.740–0.949), while participants with high fat and low lean mass had the highest cancer risk (HR = 1.207, 95% CI, 1.068–1.365).

In sensitivity analyses (Table S7), similar to the primary results, the participants in the moderately stable‐increasing and high‐stable groups had significantly reduced cancer risk. In addition, we adjusted for the predicted lean mass in 2010, and the results remained robust (Table S7). The results of the competitive risk analysis indicated that after excluding the impact of competitive events, the risk in moderately stable‐increasing and high‐stable groups remained low (Table S8).

Finally, we investigated the association between the trajectory of predicted lean mass and cancer‐specific and all‐cause mortalities (Table 4). Compared with the low‐stable group, the risk of cancer‐specific mortality was reduced by 25.4% (8.8–38.9%), 36.5% (20.3–49.4%) and 35.4% (17.9–49.2%), and the risk of all‐cause mortality was reduced by 24.2% (16.9–30.8%), 37.0% (30.0–43.2%) and 47.4% (41.0–53.1%) in the low‐increasing, moderately stable‐increasing and high‐stable groups, respectively.

**Table 4:** Association of predicted lean mass trajectories with cancer specific mortality

Trajectory pattern	All‐cause mortality				Cancer‐specific mortality			
	Mortality rate^a^	No. of deaths	HR (95% CI)^b^	*P*	Mortality rate^a^	No. of deaths	HR (95% CI)^b^	*P*
Total	8.54	4017			1.91	2183		
Low stable	11.78	1480	Ref.		2.40	307	Ref.	
Low increasing	8.52	725	0.758 (0.692, 0.831)	<0.001	1.77	151	0.746 (0.611, 0.912)	0.004
Moderately stable: Decreasing	10.14	505	0.840 (0.751, 0.940)	0.002	2.48	124	1.058 (0.839, 1.334)	0.635
Moderately stable: Increasing	7.11	611	0.630 (0.568, 0.700)	<0.001	1.50	128	0.635 (0.506, 0.797)	<0.001
High stable	6.21	768	0.526 (0.469, 0.590)	<0.001	1.54	188	0.646 (0.508, 0.821)	<0.001

^a^ Mortality rate were presented as per 1000 person‐years.

^b^ Model was adjusted for age, predicted fat mass in 2010, gender, BMI, sedentary, physical activity, smoke, alcohol use, salt consumption, high‐fat diet, hs‐CRP, Scr, family history of tumour, hypertension, and diabetes mellitus.

## Discussion

In this large‐scale prospective periodic survey study, we described five distinct trajectories of predicted lean mass based on sex‐specific quintiles and found that the trajectories of predicted lean mass changes were significantly associated with cancer risk and mortality. Compared with the low‐stable group, the moderately stable‐decreasing, moderately stable‐increasing and high‐stable groups all had significantly lower cancer risks and mortality rates, especially for digestive system cancers (gastric cancer and colorectal cancer) and lung cancer. Additional analyses also showed that the baseline level of predicted lean mass was closely associated with cancer risk, and participants in quintile 5 had lower risks of digestive system and lung cancers than those in quintile 1.

Changes in anthropometric parameters, including weight, BMI, waist circumference, waist‐to‐hip ratio, FM, and FFM, reflect an individual's nutritional status to some extent and can, therefore, greatly predict the risk and prognosis of chronic diseases, including cancer.^17^, ^18^ Similar to our partial results, some studies based on the UK Biobank have also shown that FFM is strongly associated with the risk of all cancers, including lung and digestive system cancers.^7^, ^19^, ^20^ Another study on body composition using dual‐emission X‐ray absorptiometry also demonstrated a significant association between FFM and the malignancy of prostate cancer.^21^ At the same time, there are also some heterogeneous conclusions. A cross‐sectional study by Mathew et al. showed that FFM was associated with non‐Hodgkin's lymphoma and melanoma but not with other types of cancers.^22^ However, these studies are limited, and as far as we know, the lean mass changes significantly with aging, exercise and diet.^23^, ^24^ Therefore, predicting the risk and prognosis of diseases during a 10‐year or longer follow‐up period through a single measurement of indicators will undoubtedly increase the bias of predictive effects. Building trajectories of lean mass and exploring their associations with cancer risk and prognosis may be more reliable.

Interestingly, although the HR values of cancer risk for the other four groups were all less than 1 compared with the low‐stable group, there were still differences between the groups, especially for the moderately stable‐decreasing and moderately stable‐increasing groups. In the initial survey (2006), the lean mass of the two groups of participants was basically the same; however, over time and with changes in lean mass, the cancer risk of the increasing group of patients significantly decreased by approximately 20%, whereas the cancer risk of the decreasing group of participants did not show any statistical difference. This trend was observed in overall cancer risk and some specific‐site cancers. Even in patients whose lean mass was initially low, their subsequent cancer risk decreased once their lean mass increased over time. It is worth noting that similar trends were observed for cancer‐specific mortality. Compared with the low‐stable group, the moderately stable‐decreasing group did not show a decrease in cancer‐specific mortality, whereas the other three groups had varying degrees of risk reduction, which has not been described before. These results further demonstrate the importance of exploring the trajectories of lean mass, cancer risk and mortality. A single measurement of body composition has significance; however, periodic monitoring of lean mass is essential.

In the interaction and subgroup analyses, we found that besides factors such as age and physical activity that directly affect muscle function, there were also significant interactions between the predicted lean mass trajectory and lifestyle habits such as smoking and drinking. In the same lean mass trajectory, smokers' muscles did not seem to significantly reduce cancer risk compared with those of non‐smokers. We assumed that this difference was due to differences in muscle quality. Similar to a basic study by Wang et al., the results showed that muscle strength and protein synthesis signalling were reduced in mice after cigarette smoke exposure, but muscle mass was stable.^25^ Further in vivo and in vitro studies revealed the role of inflammation. Impairment of muscle function may be related to the systemic release of the proinflammatory cytokines TNF‐α and adipokines produced in adipose tissue, leading to mitochondrial dysfunction, decreased muscle strength and even muscle wasting.^26^, ^27^ In addition, increased levels of oxidative stress are one of the underlying mechanisms for this phenomenon.^28^ This had also been shown in drinkers, where acute and chronic alcohol consumption can cause skeletal muscle myopathy along with impairment of skeletal muscle strength, function and fatigue resistance.^29^ Alcohol may affect muscle function and performance through the following aspects: First, alcohol disrupts the balance between the anabolic and catabolic pathways by affecting signalling pathways, the imbalance of which can lead to altered muscle morphology or loss of function without timely intervention and repair.^30^, ^31^ Second, alcohol also reduces the ability of muscle to regenerate after injury, thus causing some irreversible damage.^32^, ^33^ Finally, alcohol can also cause mitochondrial dysfunction and increased oxidative stress, and these abnormalities can delay the regeneration and recovery of muscle function and contribute to alcohol‐related muscle dysfunction and metabolic pattern shifts.^32^ Altogether, the association of lean mass trajectories with cancer risk was influenced by unhealthy lifestyles.

This study has several limitations. First, the lean mass and fat mass in the present study were calculated using a well‐recognized formula, so this may overlook the effect of muscle distribution or intermuscular adipose tissue,^34^ but our results can still reflect, to some extent, how lean mass changes in individuals. Second, this equation categorizes the Asian population as ‘other races’, which could potentially reduce the accuracy of the calculated results. It is necessary to validate and refine the equation using large‐scale cohorts. Third, although we adjusted for several confounders, there are still many potential unknowns that we did not consider, such as the type of physical activity. Aerobic and anaerobic exercise are known to be differentially effective for muscle improvement^35^; meanwhile, special types of diet, especially protein diets, have a significant effect on lean mass and strength in adults.^36^ Finally, the study was limited by the cohort design, as the Kailuan cohort was dominated by male workers, so the generalizability of the results may require more cohorts and studies to validate.

## Conclusions

In summary, this study suggests that the lean mass trajectory is associated with the risk of cancer, cancer‐specific and all‐cause mortality. Sustained increases in lean mass or maintenance of a high and stable level of lean mass may significantly reduce the risk of cancer incidence, cancer‐specific and all‐cause mortality. A single measurement of body composition may underestimate these risks, and long‐term monitoring of body composition is particularly important for predicting participants' prognosis. Improving and optimizing lean mass and quality have significant implications for reducing the burden on cancer‐related public health.

## Funding

This work was supported by the National Key Research and Development Program (2022YFC2009600) to Dr. Hanping Shi.

## Conflicts of interest

The authors declare no conflicts of interest.

## Supporting information


**Table S1.** Comparative analysis of the discrimination of predicted lean mass for all‐cause mortality.
**Table S2.** The association of predicted lean mass trajectories with the risk of specific site cancer.
**Table S3.** Subgroup analyses for the hazard ratio (HR) of cancer according to trajectories of predicted lean mass from 2006 to 2010 in digestive system cancers.
**Table S4.** Subgroup analyses for the hazard ratio (HR) of cancer according to trajectories of predicted lean mass from 2006 to 2010 in lung cancer.
**Table S5.** Hazard Ratios and 95% CI of Cancer According to the Quintile of Predicted lean mass in 2006.
**Table S6.** Hazard Ratios and 95% CI of Cancer According to the body composition in 2006.
**Table S7.** Sensitivity analyses.
**Table S8.** The association of predicted lean mass trajectories with the risk of cancers in competing risk analysis.
**Figure S1.** RCS for predicted lean mass in 2006 and cancer risk.

## Data Availability

The datasets used during the current study are available from the corresponding author on reasonable request.
