# Supplementary material for: Predicted lean body mass trajectories, and cancer risk and cancer‐specific and all‐cause mortality: A prospective cohort study
Source: J Cachexia Sarcopenia Muscle. 2023 Nov 15;14(6):2916–24. doi: 10.1002/jcsm.13370 (PMC10751432; doi:10.1002/jcsm.13370)
Supplement: Supplementary file 1 — Table S1. Comparative analysis of the discrimination of predicted lean mass for all‐cause mortality. Table S2. The association of predicted lean mass trajectories with the risk of specific site cancer. Table S3. Subgroup analyses for the hazard ratio (HR) of cancer according to trajectories of predicted lean mass from 2006 to 2010 in digestive system cancers. Table S4. Subgroup analyses for the hazard ratio (HR) of cancer according to trajectories of predicted lean mass from 2006 to 2010 in lung cancer. Table S5. Hazard Ratios and 95% CI of Cancer According to the Quintile of Predicted lean mass in 2006. Table S6. Hazard Ratios and 95% CI of Cancer According to the body composition in 2006. Table S7. Sensitivity analyses. Table S8. The association of predicted lean mass trajectories with the risk of cancers in competing risk analysis. Figure S1. RCS for predicted lean mass in 2006 and cancer risk. [file JCSM-14-2916-s001.docx]

**Supplementary methods 1**

The sex-specific anthropometric prediction equations are presented as follows [1].

For men:

Fat mass (kg) = - 18.592 - 0.009× age (years)

- 0.080× height (cm) + 0.226 × weight (kg) + 0.387× WC (cm)

+ 0.080[if Mexican] - 0.188[if Hispanic] - 0.483[if Black] + 1.050[if other race]

Lean mass (kg) = 19.363 + 0.001× age (years)

+ 0.064 × height (cm) + 0.756 × weight (kg) - 0.366 × WC (cm)

- 0.066[if Mexican] + 0.231[if Hispanic] + 0.432[if Black] - 1.007[if other race]

For women:

Fat mass (kg) = 11.817 + 0.041× age (years)

- 0.199 × height (cm) + 0.610 × weight (kg) + 0.044× WC (cm)

+ 0.388[if Mexican] + 0.073[if Hispanic] - 1.187[if Black] + 0.325[if other race]

Lean mass (kg) = - 10.683 - 0.039× age (years)

+ 0.186 × height (cm) + 0.383 × weight (kg) - 0.043 × WC (cm)

- 0.359[if Mexican] - 0.059[if Hispanic] + 1.085[if Black] - 0.340[if other race]

[1] Liu M, Zhang Z, Zhou C, Ye Z, He P, Zhang Y, et al. Predicted fat mass and lean mass in relation to all-cause and cause-specific mortality. J Cachexia Sarcopenia Muscle. 2022;13(2):1064-1075.

**Supplementary methods 2 Definition of covariates**

| **Covariates*** | **Definition** |
| --- | --- |
| Sedentary | Maintain sitting position for at least 8 hours per day. |
| Regular physical activity | ≥ 3 times/week, ≥ 30 minutes/time of physical exercise is considered regular physical exercise |
| Smoke | Smoking was defined as having 1 cigarette/day at least for more than 6 months. |
| Alcohol consumer | Alcohol consumer was defined as having drunk ≥100 mL/day of alcohol lasting for more than 6 months, regardless of the type of alcohol. |
| Salt consumption | Salt consumption was self-reported and classified into three categories: low (<6 g/day), medium (6-9 g/day) or high (≥10 g/day). |
| Hypertension | Systolic blood pressure ≥ 140 mm Hg, and/or diastolic blood pressure ≥ 90 mm Hg, and/or previously diagnosed with hypertension. |
| Diabetes mellitus | Fasting blood glucose level ≥ 7.0mmoL/L, taking oral hypoglycemic drugs or insulin, or having a self-reported medical history. |
| Hepatitis B | Epidemiology, symptoms and positive hepatitis B surface antigen (HBsAg) are used to diagnose hepatitis B. The enzyme-linked immunosorbent assay was applied to detect HBsAg quantitatively with a standard operating procedure (Shanghai Kehua Bio-Engineering, KHB, Shanghai, China). |
| Liver cirrhosis, gallstones and polyps of the gallbladder | The ultrasonic examination was used to examine the abdominal region, including liver, gallbladder, pancreas and spleen of each participant after fasting for at least 8 hours by a panel of specialists. Liver cirrhosis, gallstone disease and gallbladder polyp were diagnosed by abdominal ultrasonography according to previous clinically established criteria [2,3] or through medical records from the Tangshan Medical Insurance System |
| Serum creatinine (Scr) | According to Chinese guidelines, Scr levels greater than 106umol/L in men and 97umol/L in women are considered hypercreatininemia. |

*All covariates were recorded in 2010.

[2] Saverymuttu SH, Joseph AE, Maxwell JD. Ultrasound scanning in the detection of hepatic fibrosis and steatosis. Br Med J (Clin Res Ed). 1986;292(6512):13-5.

[3] Esterson YB, Grimaldi GM. Radiologic Imaging in Nonalcoholic Fatty Liver Disease and Nonalcoholic Steatohepatitis. Clin Liver Dis. 2018;22(1):93-108.

**Supplementary methods 3 ICD-10 of cancers**

The types of cancers included: digestive system cancers [esophageal cancer (C15), gastric cancer (C16), small intestine cancer (C17), colorectal cancer (C18-C21), liver cancer (C22.0), pancreatic cancer (C25), gallbladder and extrahepatic bile duct cancer (C23-C24)], lung cancer (C34), urinary system tumors [kidney cancer (C64-C65), bladder cancer (C67)], lymphoma (C81-C89) and leukemia (C90-C96), gender specific tumors [breast cancer (C50), cervix cancer (C53), uterus cancer (C54-C55), ovarian cancer (C56), prostate cancer (C61)], and other cancers.

Table S1 Comparative analysis of the discrimination of predicted lean mass for all-cause mortality

|  | IDI | p | NRI | p |
| --- | --- | --- | --- | --- |
| Predicting Cancer Risk | | | | |
| Predicted lean mass trajectories | Ref. |  | Ref. |  |
| Predicted lean mass in 2006 | -0.046(-0.065,-0.007) | <0.001 | 0(-0.001,0) | <0.001 |
| Predicted lean mass in 2008 | -0.041(-0.056,-0.022) | <0.001 | 0(-0.001,0) | <0.001 |
| Predicted lean mass in 2010 | -0.015(-0.051,0.011) | 0.409 | -0.002(-0.003,0) | <0.001 |
| Predicting All-Cause Mortality | | | | |
| Predicted lean mass trajectories | Ref. |  | Ref. |  |
| Predicted lean mass in 2006 | -0.078(-0.084,-0.016) | <0.001 | -0.003(-0.003,0) | <0.001 |
| Predicted lean mass in 2008 | -0.109(-0.123,-0.004) | <0.001 | -0.002(-0.003,-0.001) | <0.001 |
| Predicted lean mass in 2010 | -0.072(-0.084,-0.016) | <0.001 | -0.002(-0.003,0) | <0.001 |
| Predicting Cancer-Specific Mortality | | | | |
| Predicted lean mass trajectories | Ref. |  | Ref. |  |
| Predicted lean mass in 2006 | -0.068(-0.089,-0.011) | <0.001 | -0.001(-0.001,0) | <0.001 |
| Predicted lean mass in 2008 | -0.078(-0.125,-0.051) | <0.001 | 0(-0.001,0) | <0.001 |
| Predicted lean mass in 2010 | -0.063(-0.082,0.046) | 0.727 | -0.001(-0.001,0) | <0.001 |

**Table S2 The association of predicted lean mass trajectories with the risk of specific site cancer**

|  | Trajectory Pattern | IR^a^ | Case^b^ | HR (95% CI) |
| --- | --- | --- | --- | --- |
| Head and neck cancer | Low Stable | 8.28 | 104 | Ref. |
|  | Low Increasing | 6.70 | 56 | 0.813(0.582,1.133) |
|  | Moderately stable - Decreasing | 10.97 | 54 | 1.412(0.987,2.019) |
|  | Moderately stable - Increasing | 7.87 | 66 | 0.963(0.683,1.356) |
|  | High Stable | 7.00 | 84 | 0.855(0.580,1.262) |
| Esophageal cancer | Low Stable | 2.39 | 30 | Ref. |
|  | Low Increasing | 1.79 | 15 | 0.730(0.386,1.378) |
|  | Moderately stable - Decreasing | 1.22 | 6 | 0.472(0.184,1.210) |
|  | Moderately stable - Increasing | 1.19 | 10 | 0.470(0.213,1.036) |
|  | High Stable | 1.25 | 15 | 0.473(0.202,1.109) |
| Gastric cancer | Low Stable | 3.90 | 49 | Ref. |
|  | Low Increasing | 3.47 | 29 | 0.830(0.518,1.329) |
|  | Moderately stable - Decreasing | 4.06 | 20 | 0.820(0.463,1.452) |
|  | Moderately stable - Increasing | 3.46 | 29 | 0.725(0.433,1.214) |
|  | High Stable | 2.50 | 30 | **0.435(0.237,0.799)** |
| Small bowel cancer | Low Stable | 0.32 | 4 | Ref. |
|  | Low Increasing | 0.48 | 4 | 1.210(0.293,4.990) |
|  | Moderately stable - Decreasing | 0.00 | 0 | NA(NA,NA) |
|  | Moderately stable - Increasing | 0.60 | 5 | 1.051(0.238,4.635) |
|  | High Stable | 0.58 | 7 | 0.643(0.121,3.414) |
| Colorectal cancer^c^ | Low Stable | 5.49 | 69 | Ref. |
|  | Low Increasing | 3.23 | 27 | **0.575(0.365,0.906)** |
|  | Moderately stable - Decreasing | 3.05 | 15 | 0.771(0.493,1.206) |
|  | Moderately stable - Increasing | 4.53 | 38 | **0.521(0.287,0.944)** |
|  | High Stable | 4.00 | 48 | 0.645(0.385,1.081) |
| Liver cancer^c^ | Low Stable | 3.82 | 48 | Ref. |
|  | Low Increasing | 2.51 | 21 | 0.643(0.381,1.086) |
|  | Moderately stable - Decreasing | 3.25 | 16 | 0.743(0.400,1.380) |
|  | Moderately stable - Increasing | 3.10 | 26 | 0.736(0.434,1.246) |
|  | High Stable | 3.59 | 43 | 0.857(0.481,1.528) |
| Gallbladder and extrahepatic cholangiocarcinoma^d^ | Low Stable | 0.56 | 7 | Ref. |
|  | Low Increasing | 0.48 | 4 | 0.714(0.202,2.529) |
|  | Moderately stable - Decreasing | 0.00 | 0 | NA(NA,NA) |
|  | Moderately stable - Increasing | 0.12 | 1 | 0.129(0.014,1.175) |
|  | High Stable | 0.42 | 5 | 0.314(0.059,1.664) |
| Pancreatic cancer | Low Stable | 1.11 | 14 | Ref. |
|  | Low Increasing | 0.84 | 7 | 0.839(0.333,2.115) |
|  | Moderately stable - Decreasing | 1.02 | 5 | 1.022(0.342,3.052) |
|  | Moderately stable - Increasing | 0.95 | 8 | 1.032(0.397,2.679) |
|  | High Stable | 1.42 | 17 | 1.578(0.585,4.260) |
| Digestive system tumors^e^ | Low Stable | 17.59 | 221 | Ref. |
|  | Low Increasing | 12.79 | 107 | **0.701(0.554,0.888)** |
|  | Moderately stable - Decreasing | 12.59 | 62 | **0.709(0.552,0.911)** |
|  | Moderately stable - Increasing | 13.94 | 117 | **0.627(0.474,0.831)** |
|  | High Stable | 13.76 | 165 | **0.614(0.453,0.833)** |
| Lung cancer | Low Stable | 14.64 | 184 | Ref. |
|  | Low Increasing | 12.43 | 104 | 0.856(0.625,1.173) |
|  | Moderately stable - Decreasing | 12.19 | 60 | 0.873(0.682,1.116) |
|  | Moderately stable - Increasing | 10.61 | 89 | **0.688(0.521,0.908)** |
|  | High Stable | 10.92 | 131 | **0.683(0.504,0.926)** |
| Breast cancer^b^ | Low Stable | 13.20 | 35 | Ref. |
|  | Low Increasing | 13.50 | 25 | 0.849(0.496,1.456) |
|  | Moderately stable - Decreasing | 9.61 | 8 | 0.599(0.271,1.324) |
|  | Moderately stable - Increasing | 12.89 | 23 | 0.670(0.371,1.210) |
|  | High Stable | 18.48 | 48 | 0.747(0.399,1.398) |
| Female reproductive system cancer ^b, e^ | Low Stable | 2.26 | 6 | Ref. |
|  | Low Increasing | 5.94 | 11 | 2.030(0.725,5.679) |
|  | Moderately stable - Decreasing | 4.80 | 4 | 1.629(0.444,5.982) |
|  | Moderately stable - Increasing | 6.17 | 11 | 1.805(0.610,5.335) |
|  | High Stable | 7.70 | 20 | 1.751(0.545,5.620) |
| Prostatic cancer ^b^ | Low Stable | 1.41 | 14 | Ref. |
|  | Low Increasing | 1.38 | 9 | 0.879(0.372,2.075) |
|  | Moderately stable - Decreasing | 1.47 | 6 | 0.704(0.249,2.042) |
|  | Moderately stable - Increasing | 1.21 | 8 | 0.613(0.231,1.627) |
|  | High Stable | 0.75 | 7 | 0.554(0.169,1.818) |
| Tumor of urinary system^e^ | Low Stable | 2.47 | 31 | Ref. |
|  | Low Increasing | 2.75 | 23 | 1.139(0.655,1.980) |
|  | Moderately stable - Decreasing | 2.44 | 12 | 0.898(0.437,1.845) |
|  | Moderately stable - Increasing | 1.91 | 16 | 0.757(0.389,1.470) |
|  | High Stable | 3.17 | 38 | 1.184(0.610,2.299) |
| Lymphoma and leukemia | Low Stable | 1.19 | 15 | Ref. |
|  | Low Increasing | 1.08 | 9 | 0.795(0.341,1.855) |
|  | Moderately stable - Decreasing | 0.81 | 4 | 0.451(0.139,1.465) |
|  | Moderately stable - Increasing | 0.72 | 6 | 0.407(0.144,1.150) |
|  | High Stable | 1.42 | 17 | 0.589(0.219,1.583) |
| Bone tumors | Low Stable | 0.64 | 8 | Ref. |
|  | Low Increasing | 0.84 | 7 | 1.297(0.460,3.659) |
|  | Moderately stable - Decreasing | 0.41 | 2 | 0.629(0.123,3.228) |
|  | Moderately stable - Increasing | 0.48 | 4 | 0.734(0.199,2.707) |
|  | High Stable | 0.58 | 7 | 0.873(0.221,3.458) |

^a^ Incidence rate were presented as per 10,000 person-years.

b The number of people in each group is 12060, 8027, 4725, 8053, 11509. The breast cancer and female reproductive system cancers are women-specific. The number of people in each group is 2544, 1777, 799, 1712, 2493. The prostate cancer is men-specific. The number of people in each group is 9516, 6250, 3926, 6431, 9016.

c We additionally adjusted for cirrhosis and hepatitis B for digestive system cancer.

d We additionally adjusted for ALT, gallbladder stones and polyps in patients with liver cancer, gallbladder, and extrahepatic bile duct cancers.

e Digestive system cancers include esophageal cancer, gastric cancer, colorectal cancer, liver cancer, gallbladder and extrahepatic bile duct cancer, pancreatic cancer, and female reproductive system cancers include cervical cancer, endometrial cancer, and ovarian cancer; Urinary system cancers include kidney cancer and bladder cancer.

**Table S3 Subgroup analyses for the hazard ratio (HR) of cancer according to trajectories of predicted lean mass from 2006 to 2010 in digestive system cancers**

|  | N | Low Stable | Low Increasing | Moderately stable - Decreasing | Moderately stable - Increasing | High Stable | P for interaction |
| --- | --- | --- | --- | --- | --- | --- | --- |
| Sex |  |  |  |  |  |  | 0.588 |
| Women | 9325 | Ref. | 0.627(0.285,1.381) | 0.219(0.047,1.022) | 0.671(0.301,1.492) | 0.486(0.186,1.268) |  |
| Men | 35046 | Ref. | 0.683(0.532,0.877) | 0.656(0.481,0.896) | 0.694(0.534,0.902) | 0.627(0.471,0.833) |  |
| Age (years) |  |  |  |  |  |  | 0.824 |
| <45 | 10312 | Ref. | 0.788(0.314,1.977) | 0.808(0.258,2.528) | 0.824(0.336,2.017) | 0.463(0.168,1.277) |  |
| ≥45 | 34062 | Ref. | 0.692(0.541,0.885) | 0.607(0.444,0.830) | 0.709(0.547,0.919) | 0.710(0.537,0.940) |  |
| Regular PA |  |  |  |  |  |  | 0.053 |
| No | 38090 | Ref. | 0.671(0.517,0.870) | 0.668(0.482,0.924) | 0.703(0.536,0.921) | 0.589(0.437,0.795) |  |
| Yes | 6284 | Ref. | 0.789(0.444,1.400) | 0.439(0.187,1.032) | 0.700(0.371,1.319) | 0.854(0.443,1.645) |  |
| Sedentary time |  |  |  |  |  |  | 0.718 |
| <8 | 34136 | Ref. | 0.680(0.524,0.882) | 0.637(0.457,0.887) | 0.688(0.524,0.905) | 0.633(0.471,0.852) |  |
| ≥8 | 10238 | Ref. | 0.737(0.419,1.298) | 0.549(0.255,1.181) | 0.764(0.417,1.398) | 0.608(0.311,1.189) |  |
| Obesity |  |  |  |  |  |  | **0.045** |
| No | 17201 | Ref. | 0.748(0.553,1.011) | 0.811(0.468,1.405) | 0.457(0.278,0.752) | 0.473(0.207,1.078) |  |
| Yes | 27173 | Ref. | 0.662(0.447,0.981) | 0.632(0.425,0.938) | 0.851(0.604,1.198) | 0.680(0.479,0.964) |  |
| Smoke |  |  |  |  |  |  | **0.012** |
| No | 28919 | Ref. | 0.601(0.420,0.861) | 0.543(0.336,0.877) | 0.428(0.279,0.659) | 0.559(0.364,0.859) |  |
| Yes | 15455 | Ref. | 0.752(0.549,1.031) | 0.680(0.459,1.007) | 0.914(0.669,1.278) | 0.659(0.463,0.938) |  |
| Alcohol use |  |  |  |  |  |  | **0.004** |
| No | 27433 | Ref. | 0.672(0.484,0.933) | 0.712(0.481,1.055) | 0.880(0.639,1.212) | 0.809(0.567,1.153) |  |
| Yes | 16941 | Ref. | 0.699(0.496,0.985) | 0.538(0.334,0.865) | 0.496(0.334,0.741) | 0.452(0.295,0.690) |  |
| hs-CRP |  |  |  |  |  |  | 0.901 |
| <2 | 10909 | Ref. | 0.701(0.534,0.921) | 0.715(0.509,1.004) | 0.752(0.565,1.000) | 0.646(0.471,0.887) |  |
| ≥2 | 33465 | Ref. | 0.635(0.393,1.025) | 0.377(0.192,0.738) | 0.526(0.315,0.878) | 0.528(0.311,0.899) |  |

Model was adjusted for age, predicted fat mass in 2010, sex, BMI, sedentary, physical activity, smoke, alcohol use, salt consumption, high-fat diet, hs-CRP, Scr, family history of tumor, hypertension, diabetes mellitus.

**Table S4 Subgroup analyses for the hazard ratio (HR) of cancer according to trajectories of predicted lean mass from 2006 to 2010 in lung cancer**

|  | N | Low Stable | Low Increasing | Moderately stable - Decreasing | Moderately stable - Increasing | High Stable | P for interaction |
| --- | --- | --- | --- | --- | --- | --- | --- |
| Sex |  |  |  |  |  |  | **0.001** |
| Women | 9325 | Ref. | 0.975(0.427,2.228) | 0.810(0.247,2.651) | 1.147(0.467,2.645) | 1.652(0.664,1.408) |  |
| Men | 35046 | Ref. | 0.842(0.650,1.091) | 0.848(0.612,1.175) | 0.658(0.490,0.884) | 0.606(0.437,0.884) |  |
| Age (years) |  |  |  |  |  |  | **0.019** |
| <45 | 10312 | Ref. | 0.899(0.303,2.669) | 0.595(0.116,3.054) | 0.734(0.232,2.324) | 0.438(0.113,1.695) |  |
| ≥45 | 34062 | Ref. | 0.866(0.676,1.109) | 0.845(0.628,1.137) | 0.753(0.581,0.977) | 0.844(0.671,0.996) |  |
| Regular PA |  |  |  |  |  |  | 0.060 |
| No | 38090 | Ref. | 0.844(0.644,1.105) | 0.797(0.565,1.127) | 0.682(0.502,0.927) | 0.626(0.445,0.879) |  |
| Yes | 6284 | Ref. | 0.747(0.411,1.357) | 0.727(0.344,1.537) | 0.686(0.355,1.324) | 0.934(0.474,1.838) |  |
| Sedentary time |  |  |  |  |  |  | 0.525 |
| <8 | 34136 | Ref. | 0.895(0.679,1.178) | 0.769(0.533,1.109) | 0.682(0.499,0.933) | 0.707(0.503,0.993) |  |
| ≥8 | 10238 | Ref. | 0.774(0.446,1.345) | 1.132(0.606,2.116) | 0.768(0.417,1.414) | 0.652(0.328,1.298) |  |
| Obesity |  |  |  |  |  |  | 0.237 |
| No | 17201 | Ref. | 0.697(0.501,0.970) | 0.808(0.463,1.410) | 0.591(0.372,0.939) | 0.519(0.224,0.995) |  |
| Yes | 27173 | Ref. | 1.214(0.801,1.840) | 1.040(0.671,1.612) | 0.954(0.633,1.439) | 1.052(0.696,1.591) |  |
| Smoke |  |  |  |  |  |  | 0.603 |
| No | 28919 | Ref. | 0.896(0.649,1.237) | 0.829(0.548,1.252) | 0.657(0.454,0.951) | 0.679(0.457,1.001) |  |
| Yes | 15455 | Ref. | 0.834(0.569,1.229) | 0.917(0.563,1.494) | 0.862(0.562,1.322) | 0.859(0.531,1.389) |  |
| Alcohol use |  |  |  |  |  |  | 0.773 |
| No | 27433 | Ref. | 0.732(0.537,0.998) | 0.666(0.449,0.987) | 0.606(0.429,0.859) | 0.624(0.429,0.908) |  |
| Yes | 16941 | Ref. | 1.116(0.743,1.676) | 1.217(0.721,2.053) | 1.017(0.636,1.624) | 0.997(0.590,1.684) |  |
| hs-CRP |  |  |  |  |  |  | 0.151 |
| <2 | 10909 | Ref. | 0.906(0.678,1.210) | 0.865(0.593,1.261) | 0.819(0.590,1.138) | 0.871(0.606,1.254) |  |
| ≥2 | 33465 | Ref. | 0.823(0.515,1.316) | 0.879(0.495,1.562) | 0.639(0.370,1.100) | 0.645(0.356,1.167) |  |

Model was adjusted for age, predicted fat mass in 2010, sex, BMI, sedentary, physical activity, smoke, alcohol use, salt consumption, high-fat diet, hs-CRP, Scr, family history of tumor, hypertension, diabetes mellitus.

Figure S1 RCS for predicted lean mass in 2006 and cancer risk


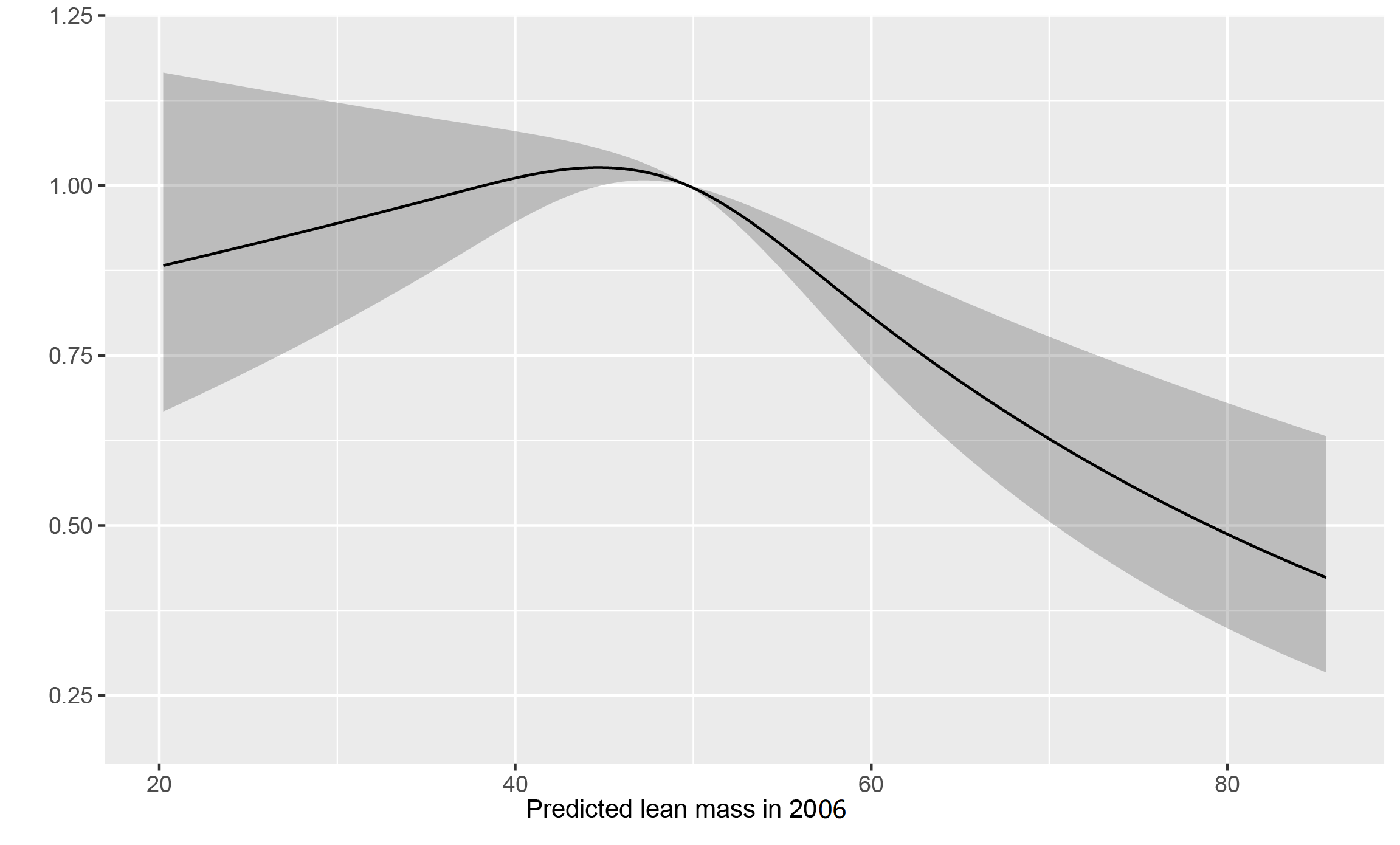


Model was adjusted for age, predicted fat mass in 2006, sex, BMI, sedentary, physical activity, smoke, alcohol use, salt consumption, high-fat diet, hs-CRP, Scr, family history of tumor, hypertension, diabetes mellitus.

**Table S5 Hazard Ratios and 95% CI of Cancer According to the Quintile of Predicted lean mass in 2006**

| Type | N | Q1 | Q2 | Q3 | Q4 | Q5 | P for trend |
| --- | --- | --- | --- | --- | --- | --- | --- |
| Head and neck cancer | 304 | Ref. | 1.146(0.824,1.592) | 1.282(0.919,1.789) | 0.883(0.601,1.298) | 1.007(0.654,1.550) | 0.878 |
| Esophageal cancer ^a^ | 56 | Ref. | 0.690(0.368,1.292) | 0.462(0.462,0.953) | 0.332(0.332,0.770) | 0.285(0.111,0.733) | **0.006** |
| Gastric cancer ^a^ | 157 | Ref. | 0.527(0.323,0.861) | 0.372(0.212,0.650) | 0.654(0.388,1.102) | 0.389(0.203,0.745) | **0.031** |
| Small bowel cancer ^a^ | 20 | Ref. | NA | 1.508(0.130,17.511) | 1.916(0.179,20.548) | 2.483(0.220,28.025) | 0.569 |
| Colorectal cancer ^a^ | 197 | Ref. | 0.893(0.582,1.371) | 0.755(0.473,1.200) | 0.776(0.472,1.274) | 0.563(0.310,1.024) | 0.072 |
| Liver cancer ^a,b^ | 154 | Ref. | 0.729(0.447,1.190) | 0.534(0.309,0.925) | 0.754(0.435,1.306) | 0.701(0.376,1.308) | 0.177 |
| Gallbladder and extrahepatic cholangiocarcinoma ^a,b^ | 17 | Ref. | 0.516(0.119,2.242) | 0.443(0.094,2.097) | 0.355(0.064,1.966) | 0.312(0.045,2.157) | 0.197 |
| Pancreatic cancer | 51 | Ref. | 0.579(0.229,1.462) | 0.706(0.277,1.794) | 0.751(0.271,2.082) | 1.421(0.483,4.186) | 0.111 |
| Digestive system tumors ^a,c^ | 672 | Ref. | 0.718(0.570,0.923) | 0.534(0.413,0.691) | 0.639(0.491,0.832) | 0.522(0.383,0.710) | **<0.001** |
| Lung cancer | 568 | Ref. | 0.893(0.699,1.142) | 0.728(0.556,0.952) | 0.690(0.515,0.924) | 0.631(0.450,0.886) | **0.004** |
| Breast cancer^b^ | 139 | Ref. | 0.484(0.272,0.860) | 0.479(0.261,0.865) | 0.566(0.305,1.051) | 0.514(0.229,1.153) | **0.050** |
| Female reproductive system cancer ^c^ | 52 | Ref. | 1.008(0.303,3.351) | 1.784(0.595,5.351) | 1.610(0.505,2.138) | 1.613(0.396,6.514) | 0.811 |
| Prostatic cancer ^b^ | 44 | Ref. | NA | NA | NA | NA | NA |
| Urinary system cancers ^c^ | 120 | Ref. | 0.987(0.553,0.903) | 0.781(0.413,0.691) | 0.753(0.378,0.832) | 1.089(0.522,1.710) | 0.651 |
| Lymphoma and leukemia | 51 | Ref. | 1.349(0.538,3.381) | 0.958(0.353,2.600) | 0.647(0.210,1.991) | 0.860(0.266,2.779) | 0.734 |
| Bone tumors | 28 | Ref. | 0.463(0.138,1.533) | 0.488(0.139,1.710) | 0.673(0.186,2.440) | 0.878(0.201,3.831) | 0.752 |

Model was adjusted for age, predicted fat mass in 2006, sex, BMI, sedentary, physical activity, smoke, alcohol use, salt consumption, high-fat diet, hs-CRP, Scr, family history of tumor, hypertension, diabetes mellitus.

^a^ We additionally adjusted for cirrhosis and hepatitis B for digestive system cancer.

^b^ We additionally adjusted for ALT, gallbladder stones and polyps in patients with liver cancer, gallbladder, and extrahepatic bile duct cancers.

^c^ Digestive system cancers include esophageal cancer, gastric cancer, colorectal cancer, liver cancer, gallbladder and extrahepatic bile duct cancer, pancreatic cancer, and female reproductive system cancers include cervical cancer, endometrial cancer, and ovarian cancer; Urinary system cancers include kidney cancer and bladder cancer.

**Table S6 Hazard Ratios and 95% CI of Cancer According to the body composition in 2006**

| Type | **Low fat mass & Low lean mass** | **Low fat mass & High lean mass** | **High fat mass & Low lean mass** | **High fat mass & High lean mass** |
| --- | --- | --- | --- | --- |
| Head and neck cancer | Ref. | **0.789(0.573,0.984)** | 1.038(0.723,1.490) | 0.738(0.521,1.045) |
| Esophageal cancer ^a^ | Ref. | **0.312(0.121,0.807)** | 0.691(0.338,1.417) | **0.490(0.248,0.967)** |
| Gastric cancer ^a^ | Ref. | **0.471(0.245,0.906)** | 1.028(0.753,1.563) | 0.858(0.537,1.372) |
| Small bowel cancer ^a^ | Ref. | 2.090(0.370,11.829) | 3.326(0.468,23.628) | 3.581(0.627,20.454) |
| Colorectal cancer ^a^ | Ref. | **0.685(0.456,0.929)** | 0.754(0.438,1.298) | 0.697(0.445,1.092) |
| Liver cancer ^a,b^ | Ref. | 0.954(0.579,1.572) | 0.914(0.536,1.555) | 0.865(0.536,1.396) |
| Gallbladder and extrahepatic cholangiocarcinoma ^a,b^ | Ref. | NA | 0.848(0.244,2.951) | 0.612(0.227,1.656) |
| Pancreatic cancer | Ref. | 0.816(0.297,2.242) | 0.832(0.295,2.343) | 1.657(0.741,3.705) |
| Digestive system tumors ^a,c^ | Ref. | **0.702(0.539,0.814)** | 0.997(0.797,1.266) | 0.848(0.676,1.004) |
| Lung cancer | Ref. | 1.050(0.813,1.369) | **1.287(1.095,1.695)** | 0.877(0.679,1.013) |
| Breast cancer^b^ | Ref. | 1.091(0.576,2.341) | 1.310(0.685,2.506) | 1.620(0.995,2.638) |
| Female reproductive system cancer ^c^ | Ref. | 1.897(0.707,5.087) | 1.655(0.605,4.481) | 1.122(0.498,2.525) |
| Prostatic cancer ^b^ | Ref. | **0.132(0.017,0.899)** | 1.443(0.743,3.234) | 0.610(0.248,1.498) |
| Urinary system cancers ^c^ | Ref. | 0.851(0.456,1.587) | 1.227(0.733,2.227) | 0.837(0.486,1.442) |
| Lymphoma and leukemia | Ref. | 0.259(0.032,1.104) | 0.893(0.254,3.065) | 0.918(0.318,2.654) |
| Bone tumors | Ref. | 0.903(0.241,2.382) | 1.670(0.524,5.315) | 1.495(0.482,4.638) |
| All cancer types | Ref. | **0.838(0.740,0.949)** | **1.207(1.068,1.365)** | **0.853(0.739,0.984)** |

Model was adjusted for age, predicted fat mass in 2006, sex, BMI, sedentary, physical activity, smoke, alcohol use, salt consumption, high-fat diet, hs-CRP, Scr, family history of tumor, hypertension, diabetes mellitus.

^a^ We additionally adjusted for cirrhosis and hepatitis B for digestive system cancer.

^b^ We additionally adjusted for ALT, gallbladder stones and polyps in patients with liver cancer, gallbladder, and extrahepatic bile duct cancers.

^c^ Digestive system cancers include esophageal cancer, gastric cancer, colorectal cancer, liver cancer, gallbladder and extrahepatic bile duct cancer, pancreatic cancer, and female reproductive system cancers include cervical cancer, endometrial cancer, and ovarian cancer; Urinary system cancers include kidney cancer and bladder cancer.

**Table S7 Sensitivity analyses**

|  | **Low Stable** | **Low Increasing** | **Moderately stable - Decreasing** | **Moderately stable - Increasing** | **High Stable** |
| --- | --- | --- | --- | --- | --- |
| **Exclude participants with cancer diagnosed within 1st year of follow-up (n=44110)** ^a^ | | | | | |
| All cancer types | Ref. | **0.823(0.717,0.945)** | **0.804(0.675,0.958)** | **0.815(0.703,0.947)** | **0.774(0.656,0.914)** |
| Digestive system tumors | Ref. | **0.640(0.497,0.824)** | **0.530(0.381,0.737)** | **0.686(0.528,0.891)** | **0.686(0.528,0.891)** |
| Lung cancer | Ref. | 0.822(0.634,1.067) | 0.760(0.544,1.062) | **0.707(0.529,0.946)** | **0.696(0.505,0.958)** |
| Gastric cancer | Ref. | 0.712(0.428,1.184) | 0.627(0.337,1.167) | 0.633(0.371,1.070) | **0.364(0.195,0.678)** |
| Colorectal cancer | Ref. | **0.538(0.334,0.867)** | **0.508(0.275,0.938)** | 0.779(0.495,1.227) | 0.629(0.385,1.002) |
| **Excluding participants with hepatitis B virus infection (n=43161)** ^a^ | | | | | |
| All cancer types | Ref. | **0.858(0.751,0.981)** | 0.859(0.728,1.016) | **0.819(0.709,0.947)** | **0.758(0.645,0.891)** |
| Digestive system tumors | Ref. | **0.713(0.555,0.914)** | **0.577(0.417,0.797)** | **0.681(0.524,0.885)** | **0.553(0.413,0.742)** |
| Lung cancer | Ref. | 0.815(0.634,1.047) | 0.745(0.538,1.033) | **0.701(0.528,0.929)** | **0.690(0.506,0.942)** |
| Gastric cancer | Ref. | 0.965(0.539,1.390) | 0.848(0.478,1.506) | 0.726(0.430,1.227) | **0.434(0.234,0.803)** |
| Colorectal cancer | Ref. | **0.568(0.358,0.901)** | **0.489(0.265,0.904)** | 0.783(0.499,1.229) | 0.648(0.385,1.089) |
| **Excluding participants with a family history of cancer (n=42735)** ^a^ | | | | | |
| All cancer types | Ref. | **0.852(0.746,0.972)** | 0.859(0.728,1.013) | **0.784(0.678,0.907)** | **0.757(0.645,0.889)** |
| Digestive system tumors | Ref. | **0.712(0.561,0.905)** | **0.614(0.451,0.837)** | **0.689(0.533,0.890)** | **0.619(0.465,0.824)** |
| Lung cancer | Ref. | 0.817(0.635,1.051) | 0.816(0.584,1.126) | **0.646(0.485,0.863)** | **0.651(0.476,0.890)** |
| Gastric cancer | Ref. | 0.865(0.534,1.399) | 0.787(0.433,1.431) | 0.701(0.409,1.201) | **0.438(0.234,0.821)** |
| Colorectal cancer | Ref. | **0.581(0.368,0.916)** | 0.744(0.473,1.172) | **0.515(0.283,0.936)** | **0.549(0.322,0.936)** |
| **Excluding participants with abnormal Scr (n=38747)** ^a^ | | | | | |
| All cancer types | Ref. | **0.863(0.752,0.989)** | 0.953(0.802,1.132) | **0.791(0.680,0.921)** | **0.837(0.713,0.983)** |
| Digestive system tumors | Ref. | **0.672(0.524,0.865)** | **0.707(0.514,0.974)** | **0.621(0.472,0.817)** | **0.625(0.466,0.839)** |
| Lung cancer | Ref. | 0.795(0.611,1.036) | 0.914(0.656,1.272) | **0.738(0.539,0.918)** | **0.667(0.495,0.900)** |
| Gastric cancer | Ref. | 0.737(0.450,1.206) | 0.822(0.452,1.495) | **0.546(0.312,0.953)** | **0.314(0.165,0.600)** |
| Colorectal cancer | Ref. | **0.524(0.322,0.854)** | 0.583(0.314,1.085) | **0.691(0.429,0.992)** | **0.550(0.320,0.944)** |
| **Further adjusted for the Predicted lean mass in 2010** ^a^ | | | | | |
| All cancer types | Ref. | **0.847(0.755,0.948)** | 0.888(0.705,1.049) | **0.801(0.667,0.901)** | **0.764(0.626,0.878)** |
| Digestive system tumors | Ref. | **0.710(0.549,0.909)** | **0.674(0.422,0.787)** | **0.688(0.537,0.891)** | **0.633(0.532,0.886)** |
| Lung cancer | Ref. | **0.804(0.623,0.999)** | 0.817(0.624,1.142) | **0.706(0.517,0.940)** | **0.687(0.500,0.902)** |
| Gastric cancer | Ref. | 0.816(0.598,1.211) | 0.823(0.721,1.199) | **0.724(0.495,0.909)** | **0.444(0.231,0.805)** |
| Colorectal cancer | Ref. | **0.527(0.395,0.711)** | **0.568(0.420,0.574)** | 0.612(0.344,1.384) | **0.644(0.344,0.962)** |
| **Further adjusted for the Time-varying variables** ^b^ | | | | | |
| All cancer types | Ref. | **0.842(0.740,0.959)** | **0.848(0.723,0.994)** | **0.789(0.687,0.907)** | **0.752(0.649,0.872)** |
| Digestive system tumors | Ref. | **0.683(0.539,0.865)** | **0.595(0.445,0.807)** | **0.679(0.531,0.869)** | **0.595(0.458,0.773)** |
| Lung cancer | Ref. | 0.844(0.660,1.081) | 0.802(0.590,1.092) | **0.703(0.533,0.927)** | **0.712(0.533,0.951)** |
| Gastric cancer | Ref. | 0.814(0.508,1.304) | 0.831(0.447,1.447) | 0.717(0.432,1.091) | **0.440(0.248,0.780)** |
| Colorectal cancer | Ref. | **0.575(0.365,0.907)** | **0.539(0.300,0.971)** | 0.782(0.504,1.115) | **0.664(0.408,0.981)** |
| **Further correcting collinearity relationships between variables** ^c^ | | | | | |
| All cancer types | Ref. | **0.891(0.782,0.991)** | 0.978(0.832,1.149) | **0.902(0.784,0.938)** | **0.762(0.627,0.812)** |
| Digestive system tumors | Ref. | **0.723(0.570,0.916)** | **0.702(0.519,0.950)** | **0.781(0.610,0.919)** | **0.728(0.543,0.952)** |
| Lung cancer | Ref. | 0.853(0.666,1.092) | 0.903(0.660,1.236) | **0.751(0.569,0.991)** | **0.721(0.510,0.994)** |
| Gastric cancer | Ref. | 0.818(0.511,1.210) | 0.867(0.495,1.421) | **0.743(0.549,0.923)** | **0.468(0.263,0.834)** |
| Colorectal cancer | Ref. | **0.568(0.361,0.894)** | 0.563(0.311,1.018) | 0.789(0.509,1.124) | **0.672(0.426,0.964)** |

^a^ Model was adjusted for age, predicted fat mass in 2010, sex, BMI, sedentary, physical activity, smoke, alcohol use, salt consumption, high-fat diet, hs-CRP, Scr, family history of tumor, hypertension, diabetes mellitus.

^b^ Model was adjusted for age (time-varying), predicted fat mass in 2010, sex, BMI (time-varying), sedentary (time-varying), physical activity (time-varying), smoke (time-varying), alcohol use (time-varying), salt consumption (time-varying), high-fat diet (time-varying), hs-CRP (time-varying), Scr (time-varying), family history of tumor, hypertension (time-varying), diabetes mellitus (time-varying).

^c^ Model was adjusted for age ^2^, predicted fat mass in 2010, sex, BMI ^2^, sedentary, physical activity, smoke, alcohol use, salt consumption, high-fat diet, hs-CRP, Scr, family history of tumor, hypertension, diabetes mellitus.

**Table S8 The association of predicted lean mass trajectories with the risk of cancers in competing risk analysis**

|  | Predicted lean mass trajectory patterns | | | | |
| --- | --- | --- | --- | --- | --- |
|  | Low Stable | Low Increasing | Moderately stable - Decreasing | Moderately stable - Increasing | High Stable |
| SD models |  |  |  |  |  |
| All cancer types | Ref. | **0.851(0.747,0.969)** | 0.864(0.735,1.015) | **0.803(0.697,0.924)** | **0.771(0.659,0.901)** |
| Digestive system tumors | Ref. | **0.707(0.559,0.895)** | **0.629(0.465,0.852)** | **0.721(0.561,0.927)** | **0.650(0.491,0.859)** |
| Lung cancer | Ref. | 0.832(0.520,1.332) | 0.819(0.463,1.448) | 0.727(0.434,1.217) | **0.435(0.237,0.798)** |
| Gastric cancer | Ref. | **0.574(0.364,0.903)** | **0.519(0.286,0.941)** | 0.768(0.491,1.201) | 0.641(0.383,1.000) |
| Colorectal cancer | Ref. | 0.832(0.651,1.063) | 0.795(0.581,1.087) | **0.694(0.525,0.914)** | **0.693(0.512,0.940)** |
| CS models |  |  |  |  |  |
| All cancer types | Ref. | **0.859(0.755,0.977)** | 0.868(0.738,1.020) | **0.813(0.707,0.935)** | **0.787(0.675,0.917)** |
| Digestive system tumors | Ref. | **0.717(0.567,0.909)** | **0.633(0.466,0.859)** | **0.736(0.576,0.941)** | **0.669(0.512,0.876)** |
| Lung cancer | Ref. | 0.846(0.531,1.349) | 0.825(0.479,1.420) | 0.745(0.465,1.191) | 0.448(0.260,0.771) |
| Gastric cancer | Ref. | **0.584(0.371,0.920)** | **0.524(0.286,0.958)** | 0.788(0.502,1.237) | 0.664(0.397,1.112) |
| Colorectal cancer | Ref. | 0.853(0.669,1.088) | 0.815(0.598,1.110) | **0.736(0.545,0.995)** | **0.720(0.546,0.949)** |

Model was adjusted for age, predicted fat mass in 2010, sex, BMI, sedentary, physical activity, smoke, alcohol use, salt consumption, high-fat diet, hs-CRP, Scr, family history of tumor, hypertension, diabetes mellitus.
